# Real-World Clinical Utility of a Methylated DNA Biomarker Assay on Samples Collected with a Swallowable Capsule-Balloon for Detection of Barrett’s Esophagus (BE)

**DOI:** 10.3390/medicina60122052

**Published:** 2024-12-13

**Authors:** Dan Lister, Andy Fine, Shail Maheshwari, Paul S. Bradley, Kimberly Lister, Victoria T. Lee, Brian J. deGuzman, Suman Verma, Lishan Aklog

**Affiliations:** 1 Arkansas Heartburn Treatment Center, Heber Springs, AR 72543, USA; 2 Colorado Primary Health Care, Littleton, CO 80120, USA; 3 Center for Digestive Disease, Shenandoah, TX 77384, USA; 4 Savii Health, Savannah, GA 31406, USA; 5 Lucid Diagnostics Inc., New York, NY 10017, USA

**Keywords:** Barrett’s esophagus, esophageal adenocarcinoma, EsoGuard, EsoCheck, clinical utility, screening, compliance

## Abstract

*Background:* Barrett’s Esophagus (BE) is the only known precursor for esophageal adenocarcinoma (EAC). Patients with multiple risk factors for BE/EAC are recommended for screening; however, few eligible patients undergo evaluation by endoscopy. EsoGuard^®^ (EG) is a commercially available biomarker assay used to analyze esophageal cells collected non-endoscopically with EsoCheck^®^ (EC) for the qualitative detection of BE/EAC. This study evaluates the real-world clinical utility of EG on cells collected with EC in patients defined by U.S. gastroenterology societies to be at-risk for BE and EAC. *Methods:* This multi-center, observational **CL**inical **U**tility of **E**soGuard (CLUE) study enrolled screening-eligible patients as defined by the American College of Gastroenterology (ACG) and the American Gastroenterological Association (AGA). Clinical utility was evaluated by the provider decision impact of EG and additionally by assessing patient compliance outcomes with recommended follow-up testing. *Results:* There were 551 patients enrolled, with a mean age of 62.0 ± 12.4 years and 56.1% (309/551) meeting ACG guideline criteria for BE screening. EC cell collection was successful in 97.1% (535/551), among which the EG positivity rate was 27.3% (*n* = 146). The provider decision impact was high, with 100% of EG-positive patients being referred for esophagogastroduodenoscopy (EGD), while 98% of EG negative patients were not referred. Among the EG-positive patients, the overall compliance with follow-up EGD was 85.4%. *Conclusions:* Combining EC non-endoscopic esophageal cell collection with the EG biomarker assay is effective in guiding provider decision-making for the detection of BE and EAC. Patients with positive EG results demonstrate high compliance with recommended follow-up EGD.

## 1. Introduction

Barrett’s Esophagus (BE) is the direct precursor to Esophageal Adenocarcinoma (EAC), the most common form of esophageal cancer in the U.S., which has increased by six-fold between 1975 and 2001 [1]. BE and EAC have the same well-defined risk factors, but in contrast to EAC, BE can be effectively treated using endoscopic techniques such as radiofrequency or cryotherapy ablation with 80–90% success rates [2,3,4]. U.S. gastroenterology societies have published guidelines and clinical practice recommendations for the diagnosis and management of BE, which include non-endoscopic alternatives to traditional esophagogastroduodenoscopy (EGD) for BE screening [5,6]. The underlying goal of BE detection is to reduce EAC mortalities by diagnosing pre-neoplastic disease, which can then be followed by either surveillance (non-dysplastic BE) or treatment (BE with dysplasia) to effectively halt disease progression [5]. 

The American College of Gastroenterology (ACG) recommends BE screening in patients with chronic gastroesophageal reflux disease (GERD) defined as ≥5 years of at least weekly symptoms, and ≥3 of the following additional risk factors: male sex, white race, age > 50 years, tobacco smoking, obesity, and family history of BE or EAC in a first-degree relative. The American Gastroenterological Association (AGA) recommends screening patients with any three or more of the same risk factors without mandating chronic GERD as a prerequisite [5,6]. The prevalence of BE is estimated to be approximately 5% within the overall U.S. population and over 10% among North Americans with GERD [7,8]. Disease in a multi-risk factor population is estimated to be anywhere between 5 and 15%; however, given the historically low rates of BE screening, this is likely a gross underestimate [9]. The literature shows that only 10–30% of patients with chronic GERD who meet appropriate screening criteria undergo EGD, attributable to both patient and provider-related factors (e.g., invasiveness of the procedure, need for sedation, patient under-reporting of heartburn symptoms, low familiarity of primary care physicians with BE guidelines) [10,11,12]. Non-endoscopic strategies for disease detection were developed to overcome several of these obstacles and to facilitate more widespread screening of at-risk patients by shifting testing to in-office settings. This strategy involves a two-step approach: 1. administering a non-invasive, accurate, office-based triage test followed by 2. confirmatory EGD in those patients with a positive result from the triage test. 

EsoGuard^®^ (EG) is the first such test to be commercially available in the U.S. It utilizes targeted Next Generation Sequencing (NGS) of DNA, combined with a proprietary algorithm to examine the presence of aberrant methylation on two specific genes (VIM and CCNA1) for the qualitative detection of BE and EAC. The esophageal cell samples for EG analysis can be collected non-endoscopically using the 510(k)-cleared EsoCheck^®^ (EC) device. The test has been clinically validated in multiple studies, demonstrating a sensitivity of up to 92.9% and a negative predictive value (NPV) of 98.6% [13,14,15]. We now present the findings of a multi-center, prospective, observational study designed to evaluate the clinical utility of the test in a real-world population at risk for BE/EAC.

## 2. Materials and Methods

This prospective, multi-center, observational study (**CL**inical **U**tility Study of **E**soGuard^®^ on Samples Collected with EsoCheck^®^ as a Triage Test for Endoscopy to Identify Barrett’s Esophagus—**CLUE**; NCT06030180) is designed to collect real-world data from among patients at increased risk for BE/EAC according to U.S. gastroenterology societies, and who undergo EG as their first step in screening. The study methods have previously been described [16]. To assess the clinical utility of EsoGuard on cells collected with Esocheck (EG/EC), provider decision impact was measured using the concordance between EG results (positive or negative) and the provider’s decision to recommend or not recommend the patient for subsequent EGD. Information on whether patients underwent their follow-up EGD was also documented as a measure of compliance outcomes. 

Study enrollment was across eight clinical sites, with a single independent ordering provider at each location. Two of the ordering providers were gastroenterologists, one a foregut surgeon, and the remaining five were primary care providers. The primary care providers referred patients to local endoscopists when they deemed EGD to be indicated based on EG results, and the specialists performed EGDs on their own patients when deemed indicated.

The follow-up period for each patient was up to 18 weeks after the EG result became available. The tolerability of the EC cell collection was measured as the rate of successful cell collections compared with the total attempted. Only patients recommended for BE screening per ACG or AGA criteria were eligible for study participation. Additionally, those with contraindications to EC cell collection (defined in the device Instructions for Use (IFU), available upon request from https://www.luciddx.com/precancer-detection/esocheck, accessed on 1 January 2024) were excluded from participation. The study was conducted according to the guidelines of the Declaration of Helsinki and approved by the WCG Institutional Review Board (IRB tracking number 20222402). All participants signed informed consent prior to the collection of any study information or cell samples. 

*EsoGuard^®^ and EsoCheck^®^:* EsoGuard is a Laboratory-Developed Test (LDT) performed in a Clinical Laboratory Improvement Amendment (CLIA) certified, College of American Pathologists (CAP) accredited, and New York State (NYS)-licensed laboratory (LucidDx Labs, Lake Forest, CA, USA) that utilizes NGS sequencing combined with a proprietary algorithm to assess the presence of cytosine methylation at 31 different genomic locations on the Vimentin (VIM) and Cyclin-A1 (CCNA1) genes. EG results are reported in a binary fashion (positive or negative), indicating the presence or absence of methylation changes to suggest the presence of BE or disease along the full BE to EAC progression spectrum. Quantity Not Sufficient (QNS) may be reported if the cell sample has insufficient DNA for EG analysis or “unevaluable” if the sample fails quality control (QC) during laboratory processing. Any patients with QNS or unevaluable samples were given the option to repeat cell collection.

**Figure 1:**
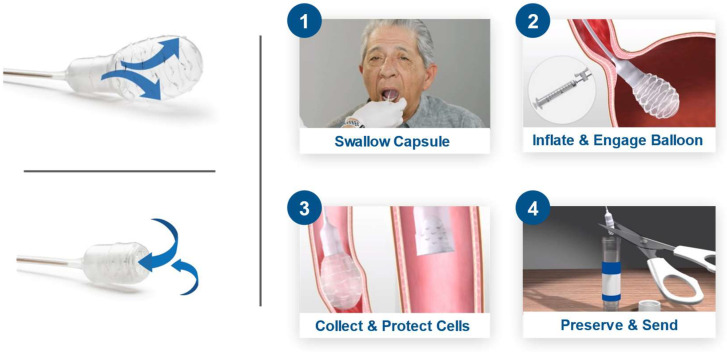
EsoCheck cell collection with Collect+Protect™ technology. Distal esophageal cell collection using the EsoCheck device: 1. Patient swallows the balloon capsule. 2. Once in the stomach, the balloon is inflated. 3. Surface esophageal cells are collected along the textured surface of the balloon, using distance markers on the silicone tubing as a guide, and the sample is protected during device retrieval by deflating the balloon back into the capsule. 4. The balloon and cell sample are shipped to the central laboratory in room temperature preservative.

EsoCheck is an FDA 510(k)-cleared swallowable ballon-capsule device designed for the non-endoscopic, circumferential, targeted sampling of mucosal cells from the distal esophagus. These cell samples can then be analyzed for cytology or with biomarker assays like EG. Cell collection can be performed in any office setting without sedation and on average takes less than five minutes (Figure 1). The unique balloon-capsule Collect+Protect™ technology ensures the specimen collected from the target region of the esophagus (distal 5 cm) is protected from contamination and dilution as it passes through the upper esophagus and oropharynx during retrieval of the device.

*Statistical Analysis:* Continuous variables are summarized using the number of observations (n), mean, standard deviation (SD), median, interquartile range (IQR), minimum, and maximum, along with the total number of patients contributing values. Categorical variables are described by the frequency of counts and percentages. The total number of applicable patients (N) is used as the denominator for percent calculations unless stated otherwise within a table footnote. Binomial exact two-sided 95% confidence intervals are calculated wherever relevant. Missing data points on BE/EAC risk factors were imputed as not present, wherever applicable.

Since provider decision impact was measured as the concordance between EG results and the decision for EGD referral/non-referral, only patients with either a positive or negative EG result were evaluated for this endpoint. Patients unable to successfully swallow the EC device and patients with QNS or unevaluable samples were included in the summary of baseline characteristics, EC tolerability, and EG results, but they did not contribute to the provider decision impact analysis.

## 3. Results

**Figure 2:**
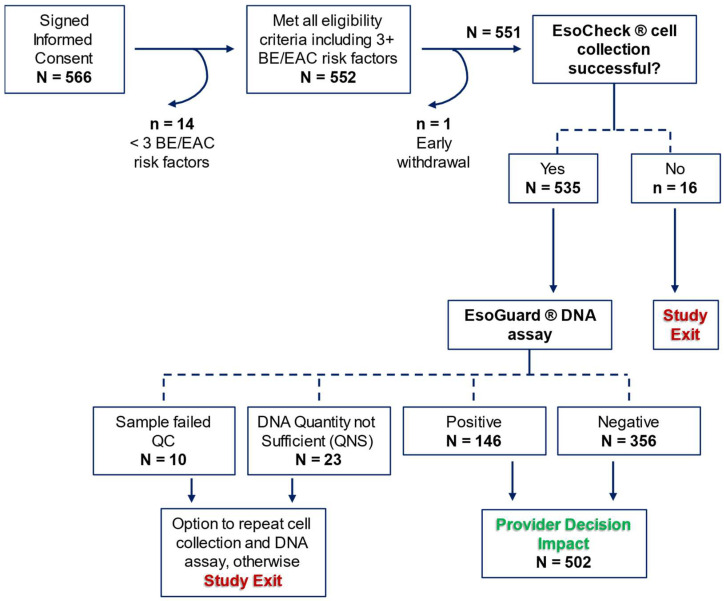
CLUE participant disposition. BE = Barrett’s Esophagus; EAC = Esophageal Adenocarcinoma; QC = Quality Control. Red text identifies when a patient could exit the study early without deviating from the protocol, and green text identifies the primary study endpoint.

There were 566 patients who consented to study participation, among which 14 were later discovered to fail eligibility criteria, and one withdrew consent prior to initiation of study procedures. Among the 551 who attempted EC cell collection, 97.1% (*n* = 535) successfully provided a sample. Binary EG results were available for 93.8% (502/535) of collected samples, and these patients contributed to the assessment of provider decision impact. Figure 2 details participant disposition in the study.

Baseline characteristics are summarized in Table 1 for the 551 patients who underwent EC. Mean age was 62.0 ± 12.4 years; 58.4% (*n* = 322) were male, 67.5% (*n* = 372) were White, 87.1% (*n* = 480) had chronic GERD, 59.9% (*n* = 330) were obese, 53.7% (*n* = 296) had tobacco smoking history (either former or current smoker), and 4.7% (*n* = 26) had a family history of BE or EAC. Patients meeting ACG risk criteria for BE and EAC i.e., the “ACG cohort” accounted for 56.1% (309/551) of study participants.

A summary of metrics for the EC cell collection and EG assay results are summarized in Table 2. EC was successful in 97.1% (535/551) of patients who attempted to swallow the device, with a median cell collection duration of four minutes. The EG positivity rate was 27.3% (*n* = 146), 4.3% (*n* = 23) of samples were QNS, and 1.9% (*n* = 10) of samples failed QC.

Patients with binary EG results were included in the assessment of provider decision impact, which is summarized in Table 3a (all evaluable patients) and Table 3b (evaluable patients meeting ACG guideline criteria for BE/EAC screening). One hundred percent of EG-positive patients were recommended/referred for EGD by their EG-ordering providers. Among the 356 patients with negative EG results, only three were referred for follow-up EGD, all of whom required the EGD for non-screening purposes. One patient underwent the EGD prior to anti-reflux surgery, and the other two patients underwent diagnostic EGD for the evaluation of intractable GERD symptoms.

Data were collected on patient follow-up among those referred for EGD after a positive EG result. Overall, 28.1% (*n* = 41) failed to undergo EGD for the reasons summarized in Table 4a (all EG-positive patients) and Table 4b (EG-positive patients meeting ACG criteria for BE/EAC screening). Among all evaluable patients, one died (0.7%) from unrelated causes before the EGD could be performed, four patients (2.7%) were referred by primary care to a gastroenterologist for EGD but were informed by the specialist that an EGD was not immediately necessary, and nine patients (6.2%) delayed the EGD due to more pressing comorbid medical conditions. Among the remaining 27 patients, nine were lost to follow-up, and the rest refused EGD (Table 4a).

Patient compliance with follow-up EGD was calculated after first excluding patients who either died before the procedure could be performed, deferred the procedure due to comorbid health issues, had an endoscopist who did not deem EGD warranted, or had incomplete data due to loss to follow-up (indicated by gray rows in Table 4a,b). Compliance was 85.4% (105/123) in the full EG-positive population and 85.1% (63/74) in the ACG cohort. Older patients within this cohort (i.e., those of Medicare-eligible age) were similarly compliant, with 85.2% (46/54) undergoing the recommended follow-up procedure.

## 4. Discussion

DNA biomarkers and non-endoscopic cell collection devices for detecting BE, such as EG and EC (respectively), may serve an important role in combating the low rates of EGD-based BE screening that has resulted in the ever-increasing incidence of EAC. We report on the clinical utility of EG/EC in real-world use across eight clinical centers, as demonstrated by provider decision impact. Additionally, we report outcome utility, measured by patient compliance with recommended EGD following a positive EG result. Our data demonstrates that EC is effective for in-office esophageal cell sample collection, and providers consistently utilize EG as a triage to EGD in patients at risk for BE and EAC who do not otherwise have an indication for urgent diagnostic upper endoscopy. Following a positive EG result, patient compliance with follow-up EGD was 85.4%, which contrasts starkly with the 10–30% of at-risk patients that typically undergo EGD for BE evaluation [10,11,12]. This data supports EC/EG as a reasonable alternative to screening EGD for more widespread and easily accessible testing in patients who meet criteria established by U.S. gastroenterology society guidelines. It may be especially useful in the primary care population to guide referrals. 

In our study population, 99.2% (353/356) of patients with negative EG results were not referred for further diagnostic workup, reflecting provider confidence in the negative predictive value of the assay. The number of EGDs that would otherwise have been required to evaluate the same volume of patients for disease was effectively reduced. There were three (3) EG-negative patients who did undergo EGD; all were for non-screening purposes (one was required prior to anti-reflux surgery, the other two for severe reflux symptoms) and not due to concern of false negative results. Among patients who were EG positive, 100% were recommended for EGD (or referred to a specialist for EGD evaluation, in the case of primary care providers). The high concordance between EG results and the ordering provider’s decision for EGD referral demonstrates strong provider decision impact. These patterns indicate that ordering providers are utilizing EG as a triage to EGD and, in doing so, can take a more intentional approach to how endoscopy resources are allocated for the purpose of diagnosing BE. 

Follow-up data showed that 85.4% (105/123) of EG-positive patients complied the recommended confirmatory EGD. The incidence of patients being “lost to follow-up” was low, at 6.2% (*n* = 9); all except for one of these patients were tested in the primary care setting. There were four patients (2.7%) referred to gastroenterologists from the primary care setting who were not scheduled for EGD based on the specialists’ judgement. All four met AGA but not ACG risk criteria for BE/EAC screening. In three of the four, the ACG guideline criteria were not met either due to insufficient chronicity of GERD symptoms (*n* = 2) or absence of GERD (*n* = 1). In the fourth individual, GERD symptoms were chronic but well-controlled on acid-suppressive medications and the patient was young (aged 39 years); other risk factors were white race and previous history of tobacco smoking. One patient died of a comorbid condition before EGD could be performed (0.7%), and nine (6.2%) deferred EGD due to comorbid health issues. These patients were not counted as “non-compliant”, given that follow-up data were either missing or the patient had indeed complied with a follow-up referral, but the endoscopist decided not to move forward. 

There were 18 patients (12.3%) who refused to comply with EGD; four of whom cited fear of the procedure and sedation, six expressed concerns about the potential cost, while the others did not clarify their reason(s) for refusal. This resulted in an overall EGD non-compliance rate of 14.6% (18/123). Non-compliance rates were similar in the cohort who met ACG guideline criteria for BE screening (14.9%; 11/74), and in the ACG sub-group aged 65 years or older (14.8%; 8/54). The >85% rate of completion for confirmatory EGDs in EG-positive patients is notably higher than typical rates of screening EGD being performed in the eligible U.S. population. Given that only 10–30% of chronic GERD patients in the literature undergo endoscopic evaluation for BE, our observed EGD compliance rates demonstrate a 2.8 to 8.5-fold increase [10,11,12]. Additionally, in a 2022 study by Kolb et al., investigators utilized web-based surveys to collect data from 472 GERD patients at three academic institutions, and only 13.2% and 5.3% reported having been recommended for and completing endoscopic BE screening, respectively [17]. Thus, compliance with screening EGD was only 40% among those who were referred, which is in stark contrast to the >85% confirmatory EGD compliance rate seen in our study following a positive EG result. 

This study has some limitations. First, factors such as race and ethnicity may be inaccurately documented since they rely on patient self-reporting, and the electronic data capture system did not allow documentation of multiple ethnic backgrounds. For purposes of counting BE/EAC risk factors, patients were documented as “White” only if they were Caucasian non-Hispanic without mixed ethnicity. Additionally, among the 480 patients who were reported to have a history of GERD, 22 (4.6%) did not provide information on the duration and/or frequency of their symptoms, and therefore, it is unknown if they met definitions for “chronic” disease. Taking a conservative approach, these patients were imputed as non-chronic when assessing who met the criteria for inclusion in the ACG analysis cohort. This could therefore have led to an underestimate of the number of patients meeting ACG guideline criteria for BE screening. Consequences are likely minimal, given the consistency of provider decisions across all study cohorts. Finally, among patients who were non-compliant with confirmatory EGD, the reason(s) for refusal was unavailable for nearly half (8/18). However, the rate of non-compliance was low compared with published studies on patient willingness to undergo EGD for BE screening, where patient surveys showed 20.4% endorsed fear of the procedure as a key barrier [17]. 

## 5. Conclusions

The goal of BE screening and early disease detection is to facilitate appropriate surveillance and treatment, given the known risk for EAC progression and its associated mortality. Improved BE detection requires more widespread testing of at-risk individuals utilizing a combination of both non-endoscopic and endoscopic strategies, as supported by existing gastroenterology society guidelines. Experience from this CLUE study demonstrates that EC is easy to implement for non-endoscopic in-office esophageal cell sampling, and the EG methylated DNA assay is effective in guiding provider decision-making. Patients with positive test results also demonstrate high compliance with recommended follow-up EGD. EC/EG may be particularly useful in the primary care setting where triaging patients to or away from EGD would allow more efficient allocation of resources.

## Figures and Tables

**Table 1:** Baseline characteristics (analysis of eligible patients only).

Characteristic	N = 551 (%)
Age, years	
Mean (SD)	62.0 (12.4)
Median (IQR)	64.0 (55.0–71.0)
Age 50+	470 (85.3%)
Age 65+	260 (47.2)
Sex	
Female	229 (41.6)
Male	322 (58.4)
Race ^§^	
Caucasian, Non-Hispanic (i.e., white race)	372 (67.5)
Hispanic	45 (8.2)
Black	127 (23.0)
American Indian or Alaskan Native	4 (0.7)
Asian	3 (0.5)
Native Hawaiian	0 (0.0)
Gastroesophageal Reflux Disease (GERD)	
Yes	480 (87.1)
No	71 (12.9)
Years with GERD symptoms	
Mean (SD)	12.2 (9.7) ^▲^
Median (IQR)	10 (5.0–15.0)
Obese ^△^	
Yes	330 (59.9)
No	221 (40.1)
Tobacco smoking	
Current	119 (21.6)
Former	177 (32.1)
Never	249 (45.2)
Missing	6 (1.1)
Family history of BE or EAC in a first-degree relative	
Yes	26 (4.7)
No	525 (95.3)
Meets ACG guideline criteria for high risk of BE/EAC (ACG cohort) ^⸸^	
Yes	309 (56.1)
No	242 (43.9)
Medicare eligible (age 65+ years) sub-group of ACG cohort ^⸸^	
Yes	154 (27.9)
No	397 (72.1)

^§^ Patient self-report; ^▲^ 458 out of 480 patients with GERD (95.4%) contributed to this datapoint; 22 GERD patients did not provide information on the duration and/or frequency of their symptoms; ^△^ Obesity was determined based on Body Mass Index (BMI) and not patient self-report; ^⸸^ ACG criteria for high risk of BE/EAC included chronic GERD (≥5 years of at least weekly symptoms or use of acid-suppressive medications) and ≥3 additional BE/EAC risk factors from among white race, male sex, age > 50 years, tobacco smoking history, obesity, family history of BE or EAC in a first degree relative. Missing datapoints for any risk factors were imputed as not present for purposes of assessing whether a patient met ACG risk criteria.

**Table 2:** Summary of EsoCheck cell collection and EsoGuard results.

**EsoCheck Procedure Summary**	N = 551 * (%)
Was EsoCheck administration fully completed?	
Yes	535 (97.1)
No	16 (2.9)
Procedure duration (minutes)	
Mean (SD)	5.4 (5.0)
Median (IQR)	4 (2.0–5.0)
Range	1.0–30.0
**EsoGuard result summary**	**N = 535 (%)**
Positive	146 (27.3)
Negative	356 (66.5)
QNS	23 (4.3)
Unevaluable/cell sample failed QC	10 (1.9)

* Includes only patients who underwent the EsoCheck cell collection; QNS = DNA quantity not sufficient for EG analysis; QC = Quality Control.

**Table 3:** (**a**) Provider decision impact—all evaluable ^△^ patients; (**b**) provider decision impact—evaluable ^△^ patients meeting ACG guideline criteria for BE/EAC risk.

**(a)**				
**EsoGuard Result**		**Recommended for EGD**	**Not recommended for EGD**	**Total**
Positive		146	0	146
Negative		3 ^§^	353	356
Total		149	353	502
Concordance between EGD referral decision and EG result (95% CI)		98.0% (95.7, 100)	100% (100, 100)	
**(b)**				
	**EsoGuard Result**	**Recommended for EGD**	**Not Recommended for EGD**	**Total**
**Full ACG cohort**	Positive	86	0	86
	Negative	1 ^†^	195	196
	Total	87	195	282
Concordance between EGD referral decision and EG result (95% CI)		98.9% (96.6, 100)	100% (100, 100)	
**Medicare-eligible** **Sub-group of ACG cohort**	Positive	60	0	60
	Negative	0	80	80
	Total	60	80	140
Concordance between EGD referral decision and EG result (95% CI)		100% (100, 100)	100% (100, 100)	

^△^ Evaluable patients are only those with binary EsoGuard results (positive or negative). ^§^ All three patients were recommended for non-screening EGD; one patient required EGD prior to anti-reflux surgery, while the other two underwent diagnostic EGD for intractable GERD symptoms. ^†^ Underwent EGD prior to anti-reflux surgery.

**Table 4:** (**a**) EsoGuard (+) patient compliance outcomes following referral for EGD – all evaluable patients; (**b**) EsoGuard (+) patient compliance outcomes following referral for EGD – evaluable patients meeting ACG guideline criteria for BE/EAC risk.

(**a**)		
**Outcome**	**N = 146**	**%**
Underwent EGD *	105	71.9%
Referred to endoscopist, but patient did not undergo EGD	41	28.1%
Patient refused ^§^	18	12.3%
Deferred due to more pressing health concerns/comorbidities ^⸸^	9	6.2%
Endoscopist did not deem EGD to be warranted	4 ^▲^	2.7%
Patient lost to follow-up	9	6.2%
Patient death (unrelated health reasons) before EGD could be performed	1	0.7%
Overall compliance	105/123 = 85.4%	
(**b**)		
**Full ACG cohort (*n* = 86)**		
Did the patient undergo an upper endoscopy procedure?		
Yes *	73.3% (63/86)	
No	26.7% (23/86)	
Why was endoscopy not performed?		
Patient refused	12.8% (11/86)	
Deferred due to health/comorbid conditions	7.0% (6/86)	
Patient lost to follow-up	7.0% (6/86)	
Overall compliance	63/74 = 85.1%	
**Medicare-eligible (aged 65 years or older) sub-group of ACG cohort (*n* = 60)**		
Did the patient undergo an upper endoscopy procedure?		
Yes *	76.7% (46/60)	
No	23.3% (14/60)	
Why was endoscopy not performed?		
Patient refused	13.3% (8/60)	
Deferred due to health/comorbid conditions	6.7% (4/60)	
Patient lost to follow-up	3.3% (2/60)	
Overall compliance	46/54 = 85.2%	

* One patient who underwent EGD was unable to fully complete the procedure due to respiratory instability; at the time of study end, this patient was pending re-scheduling of a repeat EGD. ^§^ Two (2) patients were scared to undergo EGD due to the required sedation; the remaining 10 patients did not provide additional detail on the personal reasons for refusal. ^▲^ All met AGA criteria, but none met ACG guideline criteria. ^⸸^ One of the four patients had chronic GERD but was young (<40 years), two of the four had GERD symptoms of <5 years, and one patient did not have a diagnosis of GERD; none of the four patients met ACG risk criteria for BE screening. The gray rows indicate patients for whom EGD was not performed due to factors unrelated to compliance.

## Data Availability

The data presented in this study are available on request from the corresponding author due to patient privacy.
